# Atrophy of cerebellar lobule VI and primary motor cortex in cervical dystonia - a region of interest-based study

**DOI:** 10.1007/s00702-024-02839-2

**Published:** 2024-10-07

**Authors:** Kai Grimm, Fatemeh Sadeghi, Gerhard Schön, Abdullah Okar, Mathias Gelderblom, Robert Schulz, Simone Zittel

**Affiliations:** 1 Department of Neurology, University Medical Center Hamburg-Eppendorf, Martinistr. 52, 20246 Hamburg, Germany; 2 Institute of Medical Biometry and Epidemiology, University Medical Center Hamburg-Eppendorf, Hamburg, Germany

**Keywords:** Cervical dystonia, Magnetic resonance imaging, Cerebellum, Cerebellar motor network

## Abstract

**Background:**

Recently, a network model of cervical dystonia (CD) has been adopted that implicates nodes and pathways involving cerebellar, basal-ganglia and cortico-cortical connections. Although functional changes in the cerebello-thalamo-cortical network in dystonia have been reported in several studies, structural information of this network remain sparse.

**Objective:**

To characterize the structural properties of the cerebellar motor network in isolated CD patients. This includes cerebellar lobules involved in motor processing, the dentate nucleus (DN), the thalamus, and the primary motor cortex (M1).

**Methods:**

Magnetic resonance imaging data of 18 CD patients and 18 healthy control subjects were acquired. In CD patients, the motor part of the Toronto Western Spasmodic Torticollis Rating Scale was assessed to evaluate motor symptom severity. The volume of cerebellar lobules I-VI and VIII, the DN and thalamus, and the cortical thickness (CT) of M1 were determined for a region of interest (ROI)-based quantitative analysis. Volumes/CT of these ROIs were compared between groups and associated with motor symptom severity in patients.

**Results:**

The volume of lobule VI and the CT of M1 were reduced in CD patients. The volumes of the other ROIs were not different between groups. No association was identified between the structural properties of lobule VI or M1 and the severity of CD motor symptoms.

**Conclusion:**

Atrophy within the cerebellum and M1 contributes to CD’s complex motor network pathology. Further investigations are needed to ascertain the mechanisms underlying the local volume loss.

## Introduction

Dystonia is a movement disorder characterized by sustained or intermittent muscle contractions leading to abnormal postures or movements or both (Albanese et al. 2013a). According to a recent epidemiological study, cervical dystonia (CD) is by far the most common type of isolated dystonia with a prevalence of about 40% of all cases (Dressler et al. 2022). In the past years, a network model of dystonia has been adopted that implicates nodes and pathways involving cerebellar, basal ganglia, and cortico-cortical connections (Neychev et al. 2011; Hallett et al. 2020; Giannì et al. 2022). For motor control, the basal ganglia are intricately connected to the motor cortex on one side and the cerebellum on the other in a regulatory circuit that is mediated by the thalamus (Alexander et al. 1991). Given the limited understanding of the complex networks underlying dystonia, a profound characterization of the changes that constitute the disorder remains a focus of current research in the field.

In recent years, the role of the cerebellum in the sensorimotor network in dystonia has attracted considerable interest. Studies in dystonia patients revealed that the cerebello-thalamo-cortical tract (CTC), in particular, is an important link within the motor network in dystonia. A recent magnetic resonance imaging (MRI) study that employed a connectome-based approach demonstrated abnormal connectivity of the cerebellum and the somatosensory cortex in the brain network in CD patients with and without structural brain lesions (Corp et al. 2019). Furthermore, diffusion tensor imaging (DTI) studies showed reduced structural connectivity within the CTC in idiopathic CD and genetic dystonia patients (Argyelan et al. 2009; Vo et al. 2013; Sondergaard et al. 2021, 2023). Aside from neuroimaging studies, experiments with non-invasive brain stimulation corroborated that the cerebellum is relevant in the pathophysiology of dystonia and revealed that cerebellar-brain inhibition (CBI) (Brighina et al. 2009; Porcacchia et al. 2019) and cerebellar modulation of sensorimotor integration are impaired in CD patients (Grimm et al. 2023). Moreover, the first studies revealed that cerebellar deep brain stimulation has the capability to improve motor symptoms of dystonia (Sokal et al. 2015; Diniz et al. 2021).

**Fig. 1:**
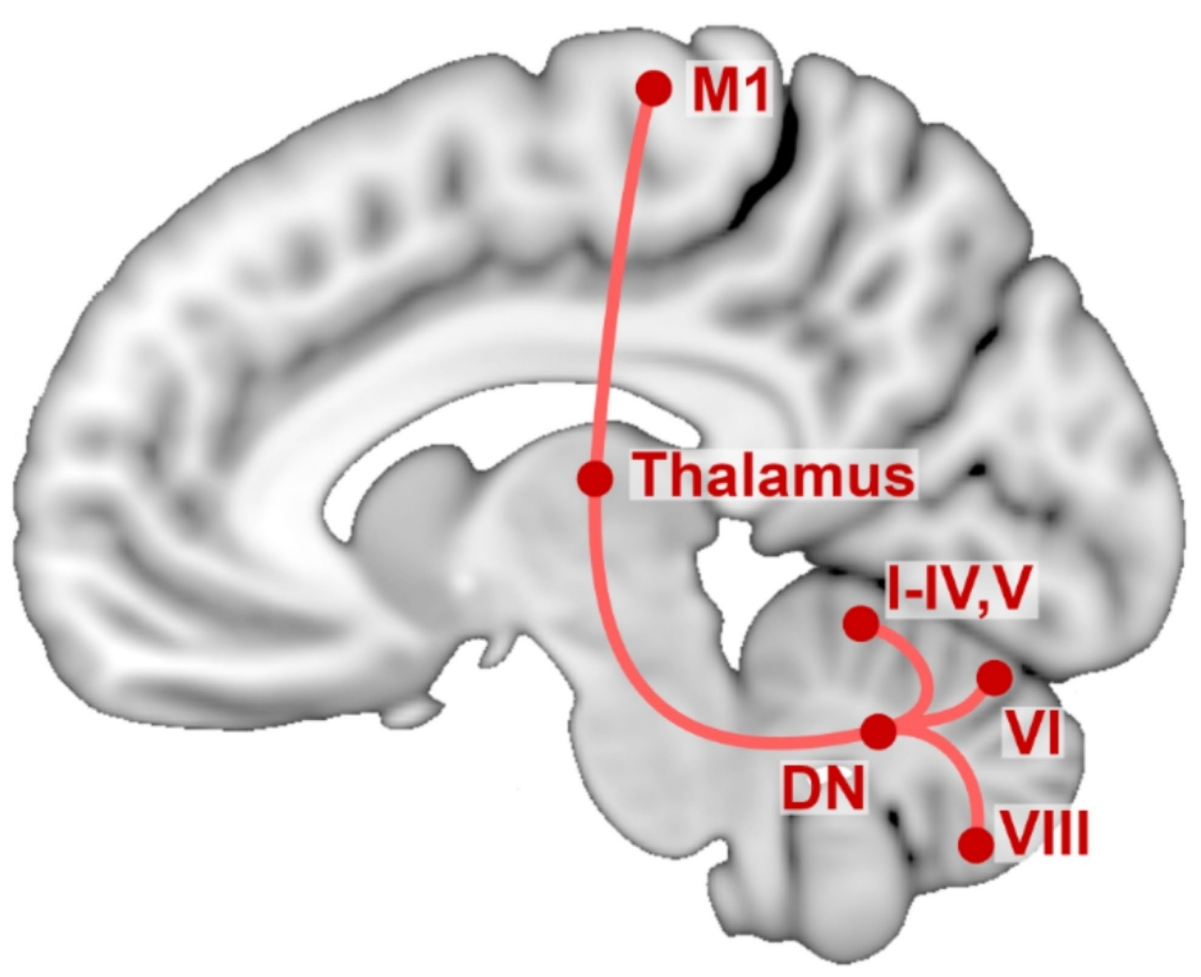
Schematic overview of the investigated regions of interest and their anatomical connections overlaid on the standardized MNI152 brain. All regions of interest are part of the cerebellar motor network. Roman numerals refer to the corresponding cerebellar lobules. DN: dentate nucleus, M1: primary motor cortex

In this exploratory study, we investigated the structural characteristics of the gray matter nodes of the cerebellar motor network. This includes the cerebellar cortex lobules associated with motor processing, i.e. lobules I–VI and VIII, the dentate nucleus (DN), the thalamus, and the primary motor cortex (M1; see Fig. 1) (Habas and Manto 2018). High-resolution structural MRI of CD patients and healthy control (HC) subjects were acquired and analyzed using a region of interest (ROI)-based approach. The association between these network structures and motor symptom severity was also evaluated.

## Materials & methods

### Participants

Eighteen patients with isolated CD were recruited from the outpatient clinic of the Department of Neurology at the University Medical Center Hamburg-Eppendorf. All patients received regular treatment with botulinum toxin injections and were investigated 9 to 12 weeks after the last injection. For comparison, a group of HC matched in age, sex and handedness was included in this study. None of the patients or healthy participants presented with any concurrent neurological disorders or was taking any centrally-acting medication.

All participants underwent a neurological examination prior to inclusion in the study. In CD patients, motor symptom severity was assessed with the motor subscore of the Toronto Western Spasmodic Torticollis Rating Scale (TWSTRS) (Albanese et al. 2013b). The clinical examination was recorded on video for blinded rating by an experienced movement disorder specialist (SZ). Demographic information, including age, sex, and, in patients, disease duration, was collected. Handedness was evaluated using the Edinburgh Handedness Inventory (Oldfield 1971). All participants completed safety questionnaires for MRI and provided written informed consent in accordance with the Declaration of Helsinki. The local ethics committee approved the study.

### MRI data acquisition

Imaging data was acquired on a 3T Siemens Magnetom Prisma MRI scanner (Siemens Healthcare, Forchheim, Germany) with a 32-channel head coil. The parameters for T1-weighted magnetization-prepared rapid gradient echo (MPRAGE) sequences were: echo time (TE) = 2.15 ms, repetition time (TR) = 2500 ms, inversion time (TI) = 1100 ms, flip angle = 8°, voxel resolution = 0.8 × 0.8 × 0.8 mm. The susceptibility-weighted image (SWI) acquisition parameters were TE = 20 ms, TR = 27 ms, flip angle = 15°, and voxel resolution = 0.8 × 0.8 × 1.2 mm.

### Segmentation of regions of interest

Cerebellar cortex segmentation was conducted as described in a previous work (Sadeghi et al. 2023). To summarize, T1-weighted MRI images were visually inspected to exclude artifacts and unexpected pathology. Then, the images were automatically processed using the CERES pipeline, which performs segmentation, denoising, cropping, inhomogeneity correction, and different steps of registration to the MNI152 template (Romero et al. 2017). Finally, volumes of the cerebellar lobules of interest (I–VI, and VIII) were calculated in milliliters (ml). In addition to lobular volumes, the total intracranial volume (ICV) was estimated. The segmentation results were visually verified to identify outliers.

An experienced MRI investigator (KG) performed the DN segmentation manually, validated by a second independent examiner blinded to the participant group (AO). This process was performed using the segmentation tool ITK-SNAP (Yushkevich et al. 2016) in both cerebellar hemispheres. It followed a standardized workflow: The DN was identified on the SWI images by its characteristic shape resembling a sac with lobed walls. In the first iteration, the corrugated outline of the DN was traced and filled in the axial plane. During the second and third iterations, this volume was initially refined in the coronal view and subsequently in the axial view. The validation of the segmentation entailed visual inspection of the DN ROIs in the axial and coronal planes with a particular focus on consistent inclusion or exclusion of distinguishable structures across subjects. If the second examiner reported a divergence from the initial segmentation, a consensus was reached for the optimized final DN segmentation.

The analysis of the thalamus volume and cortical thickness (CT) of the precentral gyrus was conducted using the Connectome Mapper 3 pipeline (v3.1.0) (Tourbier et al. 2022). The pipeline integrates the FreeSurfer image analysis suite (v7.1.1, http://surfer.nmr.mgh.harvard.edu/) for the segmentation and volumetric parcellation of the human brain based on the Lausanne 2018 atlas (Cammoun et al. 2012). This atlas is an extended and enhanced version of the Desikan-Killiany atlas that includes probabilistic atlas-based segmentation of the thalamus (Najdenovska et al. 2018). The precentral gyrus was used as an approximation of M1 and will be referred to as such in the following. Visual output inspection was conducted to ensure the accurate execution of the segmentation process.

### Statistical analysis

Of the segmented cerebellar lobules, only those involved in motor processing (I, II, III, IV, V, VI, VIIIA, VIIIB) were included in the analysis (Fig. 1). Lobules I–IV were combined to a single ROI. The volume of these cerebellar lobes, the DN and the thalamus were included in the analysis in ml. M1 structure was operationalized with average CT (Hutton et al. 2009). Volumes and cortical thickness from the left and right side were averaged for the analysis.

First, we compared the volumes and CT of the ROIs across both groups using linear regression models. The models were fitted for each ROI with VOLUME or CT as the dependent and GROUP (two levels: CD and HC) and AGE as the independent variables; ICV was included as nuisance variable, except for the analysis of M1 CT where ICV was omitted. Significance of group differences and age effects was tested with likelihood ratio tests of the full model against a reduced model that did not include the target variable GROUP or AGE. A leave-one-out model analysis (LOOA) was performed to test the robustness of the results. For the ROI with significant group differences (cerebellar lobule VI and M1), we tested whether the structural properties of one ROI were associated with the structural properties of the other ROI. A linear model was fitted with the VOLUME of lobule VI as the dependent and the CT of M1 as the independent variable; GROUP and AGE were included as nuisance variables. Statistical significance of the relationship was tested with a likelihood ratio test between model variants with and without the inclusion of M1 CT. Second, we tested whether there was a relationship between structural properties of the ROIs with significant group differences and motor symptom severity in CD patients. Linear models were fitted for each ROI with TWSTRS as the dependent and VOLUME / CT as the independent variables; again, AGE and ICV were included as nuisance variables, except for the analysis of M1 CT where ICV was omitted. Statistical significance of the relationship was tested with likelihood ratio tests between model variants with and without the inclusion of VOLUME / CT and a LOOA was performed to test model robustness. P-values of < 0.05 were considered significant.

## Results

### Demographic and clinical data

The median age was 59 [range: 44–76] years in the CD cohort and 59 [43–78] years in the HC group. Eight male and ten female subjects were included in each group. In each group, one subject was ambidextrous while all other subjects were right-handed. In the CD cohort, median disease duration was 9 [1–20] years, and median motor symptom severity assessed with the TWSTRS was 18 [4–23] points (Table 1).

**Table 1:** Demographic and clinical data of the cervical dystonia patients. Disease duration refers to the number of years the patients suffered from cervical dystonia. F: female, M: male, TWSTRS: motor subscore of the Toronto Western Spasmodic Torticollis Rating Scale

Subject	Age	Sex	Disease duration	TWSTRS
1	58	M	15	4
2	54	M	10	8
3	44	F	9	21
4	73	M	20	21
5	60	M	20	21
6	61	F	2	20
7	45	F	2	15
8	50	F	11	8
9	66	M	5	12
10	51	F	9	21
11	59	F	1	20
12	65	M	4	23
13	59	F	14	18
14	44	F	9	7
15	54	F	10	12
16	60	F	5	9
17	76	M	8	17
18	66	M	8	22

### ROI structure and relationship with motor symptom severity

**Fig. 2:**
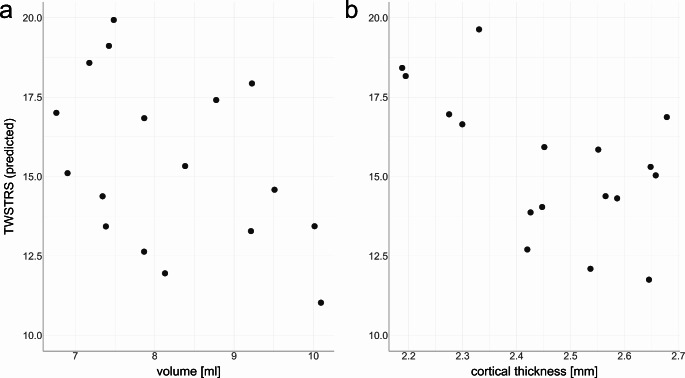
(a) Plotted values of VOLUME of lobule VI and model prediction of TWSTRS in the CD patients. The effect of VOLUME on TWSTRS was not significant (*p* = 0.291). (b) Plotted values of CT of M1 and model prediction of TWSTRS in the CD patients. The effect of CT of M1 on TWSTRS was not significant (*p* = 0.744). TWSTRS: motor subscore of the Toronto Western Spasmodic Torticollis Rating Scale, CD: cervical dystonia, CT: cortical thickness, M1: primary motor cortex

The volume of lobule VI and the CT of M1 were significantly smaller in the CD group. The volume of lobule VI was 8.21 ml [95% confidence interval: 7.65–8.78] in CD patients compared to 9.09 ml [8.53–9.65] in HC (*p* = 0.022). CT of M1 was 2.46 mm [2.40–2.53] in CD and 2.58 mm [2.52–2.65] in HC (*p* = 0.007). These results remained significant in the LOOA. Lobule V showed a trend towards smaller volume in CD patients, but the result did not reach statistical significance (*p* = 0.082). There were no differences with regard to the volumes of the other ROIs that were included in the analysis (Table 2). Age was not associated with the volumes of lobule VI (*p* = 0.242) or the CT of M1 (*p* = 0.112) and the individual volumes of lobule VI and the CT of M1 showed no significant association (*p* = 0.595). There was no effect of VOLUME / CT on TWSTRS for lobule VI (*p* = 0.291) or M1 (*p* = 0.744; see Fig. 2).

**Table 2:** Linear model estimates and 95% confidence intervals for the volumes of the investigated regions of interest (or cortical thickness in the case of M1) in both groups. The p-values for the linear model effect of GROUP are given. ROI: region of interest, CD: cervical dystonia, HC: healthy controls, M1: primary motor cortex

ROI	CD		HC		*p*-value
	Estimate	95% CI	Estimate	95% CI	
I-IV	2.69	2.54–2.84	2.70	2.55–2.84	0.98
V	3.68	3.43–3.93	3.98	3.72–4.23	0.082
VI	8.21	7.65–8.78	9.09	8.53–9.65	0.022
VIIIA	5.77	5.42–6.12	5.79	5.44–6.14	0.941
VIIIB	3.82	3.56–4.09	3.82	3.55–4.08	0.965
dentate	0.96	0.89–1.04	1.03	0.96–1.11	0.181
thalamus	7.19	6.94–7.44	7.34	7.09–7.58	0.367
M1	2.46	2.40–2.53	2.58	2.52–2.65	0.007

## Discussion

In this explorative ROI-based quantitative analysis of the gray matter nodes of the cerebellar motor network, the volume of lobule VI and CT of M1 were reduced in CD patients. No group differences were found for the volumes of the other ROIs. The structural properties of lobule VI and M1 were not associated with motor symptom severity in CD.

### Volume reduction of lobule VI in CD

In this cohort of isolated CD patients, a significant volume reduction of lobule VI was observed. These findings corroborate the results of a study by Pontillo and colleagues which is the only other study that has investigated cerebellar volume in CD with a ROI-based approach (Pontillo et al. 2020). In the referenced study of 27 CD patients, the cerebellum was segmented into lobes and lobules. The authors reported volume reductions in the anterior lobe, in the combined ROI for lobules I-IV, in lobule V, and in lobule VI. Interestingly, the effect size was larger in the present study with an estimated volume reduction of 9.7% in lobule VI compared to 4.4% observed by Pontillo and colleagues. This difference may be due to a longer disease duration in our cohort (mean duration was 9 years as opposed to 7.1 years in the study by Pontillo et al.) or to the more refined segmentation approach applied in the present study with the CERES as opposed to the SUIT segmentation pipeline (Carass et al. 2018).

Most previous studies that investigated cerebellar structure in CD patients used voxel-based morphometry (VBM). Out of twelve VBM studies (Draganski et al. 2003; Obermann et al. 2007; Egger et al. 2007; Pantano et al. 2011; Prell et al. 2013; Piccinin et al. 2014; Delnooz et al. 2015; Bono et al. 2015; Waugh et al. 2016; Filip et al. 2017; Burciu et al. 2017; Gracien et al. 2019), four reported localized increases in grey matter density within the cerebellum in CD, but the location of structural changes varied between studies (Draganski et al. 2003; Obermann et al. 2007; Piccinin et al. 2014; Filip et al. 2017). In the remaining eight studies, no gray matter changes were detected in the cerebellum of CD patients. Overall, previous results of cerebellar analysis with VBM were highly heterogeneous.

In the cerebellum, motor functions are represented in the lobules I-VI and VIII (Manni and Petrosini 2004; Guell and Schmahmann 2020). These motor areas are organized somatotopically with lobule VI representing the body regions of the neck and the face (Manni and Petrosini 2004; Mottolese et al. 2013). Therefore, the present results reveal a local atrophy in the cerebellar motor regions corresponding to the body area affected in CD. The underlying anatomical substrate for this local volume loss remains speculative. Results from previous studies suggest that the atrophy of lobule VI might be due to a loss of Purkinje cells. First, in a neuropathological report of six CD patients and 16 HC subjects, Prudente and co-workers reported a patchy loss of Purkinje cells, areas of focal gliosis, and torpedo bodies in the cerebellum (Prudente et al. 2013). This finding has to be considered with caution because Purkinje cell loss is not specific to dystonia but can also be found in other degenerative and non-degenerative neurological conditions (Prudente et al. 2013). Second, in previous neurophysiological studies CBI could not be evoked in CD patients (Brighina et al. 2009; Porcacchia et al. 2019). CBI is a transcranial magnetic stimulation paradigm that combines the application of a conditioning stimulus to the cerebellum followed by a test stimulus to the contralateral M1 at short interstimulus intervals of 5–7 ms (Fernandez et al. 2018). The conditioning stimulus is thought to activate Purkinje cells and thereby temporarily reduces the facilitating influence of the dentate on the thalamus and M1 (Ugawa et al. 1991, 1995).

### Reduced cortical thickness of M1 in CD

Another main finding is reduced CT of M1 in CD patients. This finding of cortical thinning of M1 in CD is in line with the results of previous studies that applied a ROI-based approach. Several studies reported a reduced CT or a volume loss of M1 in CD patients (Vilany et al. 2017; Tomić et al. 2021; Wu et al. 2022). The referenced studies that performed volumetry via VBM (Draganski et al. 2003; Obermann et al. 2007; Egger et al. 2007; Pantano et al. 2011; Prell et al. 2013; Delnooz et al. 2015; Bono et al. 2015; Waugh et al. 2016; Filip et al. 2017; Burciu et al. 2017; Gracien et al. 2019) yielded similar results: Three detected a volume reduction of M1 (Pantano et al. 2011; Prell et al. 2013; Bono et al. 2015), only one found a focal volume increase in CD (Draganski et al. 2003).

Unlike the cerebellum, M1 has not been the object of neuropathological investigations in CD and therefore it is unknown which microscopic changes underlie the CT reduction of M1 (Sharma 2019). We conjecture that reduced CT in this motor area may reflect either a local loss of neurons or a reduction in synaptic density or both. This would be consistent with previous findings from neurophysiological experiments and connectivity studies. The former showed that both intracortical inhibition (Kanovský et al. 2003; Berardelli et al. 2008), which represents short-range inhibitory circuitry, and CBI (Brighina et al. 2009; Porcacchia et al. 2019), which relies on long-range inhibitory projections, are impaired in dystonia. Alterations in cortico-cortical connectivity from the sensory cortex to M1 may also play a role, but this has not yet been investigated in dystonia (Brown et al. 2019). Furthermore, connectivity studies based on DTI reported reduced fiber integrity of the CTC in isolated CD as well as in manifesting and non-manifesting *TOR1A* and *THAP1*-mutation carriers (Argyelan et al. 2009; Vo et al. 2013; Sondergaard et al. 2021, 2023).

### No changes found in other regions of interest

No group differences were found for the other investigated ROIs, i.e. cerebellar lobules IV and VIII, the DN, and the thalamus. Previous studies reported heterogeneous findings regarding the thalamus. In three previously conducted ROI-based studies, the thalamus was smaller in CD patients (Waugh et al. 2016; Tomić et al. 2021; Wu et al. 2022). In contrast, gray matter volume increases in the thalamus of CD patients were detected in two out of the cited VBM studies, while the other studies yielded no group differences (Obermann et al. 2007; Filip et al. 2017). For the comparison of DN volume, no other ROI-based study was performed previously. Out of the eleven VBM studies, only one identified a volume reduction of the DN, while the others found no differences (Filip et al. 2017).

### Limitations

The present study has several limitations. First, the sample size is small, a common limitation in MRI studies in CD (Huang et al. 2022). As a result, the study may be underpowered to detect subtle effects that may nevertheless be relevant. Second, we focused our analysis specifically on structural changes of the cerebellar motor network. Possible changes in other parts of the complex sensorimotor network in dystonia might not have been detected. Thirdly, MRI-based studies of brain structure in dystonia cannot distinguish between morphologic alterations that are causal and those that are compensatory mechanisms.

## Conclusion

This study reveals a localized volume loss of the cerebellar lobule VI and cortical thinning of M1 in CD patients. While the cerebellar volume reduction may be due to a loss of Purkinje cells, the reasons for structural alterations of M1 remain less clear. Microstructural neuropathological studies or animal models of dystonia are required to provide a more thorough understanding of these MRI findings and to link them to neurophysiological findings with a higher degree of certainty.
